# The Pathogenic and Therapeutic Implications of Ceramide Abnormalities in Atopic Dermatitis

**DOI:** 10.3390/cells10092386

**Published:** 2021-09-10

**Authors:** Masanori Fujii

**Affiliations:** Department of Pharmacology, Division of Pathological Sciences, Kyoto Pharmaceutical University, Kyoto 607-8414, Japan; fujii@mb.kyoto-phu.ac.jp

**Keywords:** ceramide, skin barrier, atopic dermatitis

## Abstract

Ceramides play an essential role in forming a permeability barrier in the skin. Atopic dermatitis (AD) is a common chronic skin disease associated with skin barrier dysfunction and immunological abnormalities. In patients with AD, the amount and composition of ceramides in the stratum corneum are altered. This suggests that ceramide abnormalities are involved in the pathogenesis of AD. The mechanism underlying lipid abnormalities in AD has not yet been fully elucidated, but the involvement of Th2 and Th1 cytokines is implicated. Ceramide-dominant emollients have beneficial effects on skin barrier function; thus, they have been approved as an adjunctive barrier repair agent for AD. This review summarizes the current understanding of the mechanisms of ceramide abnormalities in AD. Furthermore, the potential therapeutic approaches for correcting ceramide abnormalities in AD are discussed.

## 1. Introduction

Ceramides are sphingolipids with diverse physiological roles. In the skin, ceramides and other lipids play an essential role in forming a permeability barrier in the stratum corneum (SC), preventing excessive water loss and the invasion of harmful substances from the environment [1].

Atopic dermatitis (AD) is a common chronic skin disease associated with skin barrier dysfunction and immunological abnormalities [2,3]. In patients with AD, the amount and composition of ceramides in the SC are altered. This suggests that ceramide abnormalities are involved in the pathogenesis of AD. However, the mechanism of ceramide abnormalities in this disease state is still not fully elucidated. Nevertheless, it has been shown that ceramide-dominant emollients can restore skin barrier function; thus, they have been approved as an adjunctive barrier repair agent for AD [4].

This review provides a concise but comprehensive summary of the current knowledge regarding (1) the role of ceramides in normal skin barrier formation, (2) the metabolism of ceramides in the epidermis, (3) ceramide abnormalities in AD and their possible underlying mechanisms, and (4) potential therapeutic approaches for correcting ceramide abnormalities in AD.

## 2. SC as a Permeability Barrier

**Figure 1:**
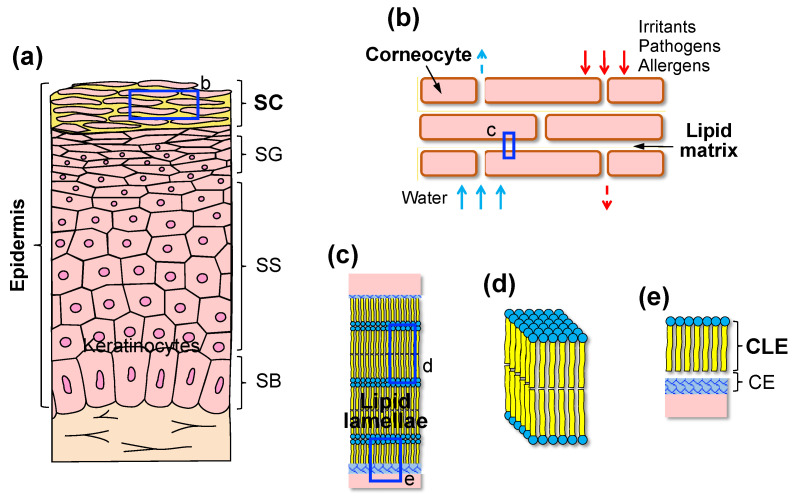
The stratum corneum (SC) as a permeability barrier. (**a**) The structure of the epidermis. The epidermis is composed of four different layers: the SC, the stratum granulosum (SG), the stratum spinosum (SS), and the stratum basale (SB). (**b**) The SC is composed of corneocytes embedded in a lipid matrix and functions as a permeability barrier. (**c**) The intercellular lipids are arranged in multiple bilayers (lamellae). (**d**) Within the lipid lamellae, lipid head groups assume a very dense, ordered orthorhombic lateral organization. (**e**) Lipids bind covalently to the glutamate residues of the cornified envelope (CE) proteins, such as involucrin and loricrin, forming a lipid monolayer called the cornified lipid envelope (CLE). Each letter-labelled blue square refers to a corresponding magnified image.

The skin is the largest organ and the primary interface between the internal and external environments. The epidermis is the outermost layer of the skin. It consists of four different layers: the superficial SC, the stratum granulosum (SG), the stratum spinosum, and the innermost stratum basale. Keratinocytes, the predominant cell type in the epidermis, proliferate in the basal layer and differentiate toward the surface (Figure 1a).

The SC provides a permeability barrier that prevents excessive water loss from the body and protects the body against environmental irritants, pathogens, and allergens [1]. The SC is composed of corneocytes, which are dead and terminally differentiated keratinocytes, embedded in an extracellular lipid matrix. The SC is often referred to as having a “brick” (corneocytes) and “mortar” (lipids) structure (Figure 1b) [5]. The SC lipids consist of an equimolar mixture of ceramides (~50% by weight), cholesterol (~25%), and free fatty acids (FFAs) (~15%), with smaller quantities of phospholipids and cholesterol sulfate [6]. These lipids are arranged in multiple bilayers called lamellae between corneocytes with a long (~13 nm) and short (~6 nm) repeat distance, which is referred to as the long periodicity phase (LPP) and the short periodicity phase (SPP), respectively [7]. The LPP is unique in the SC and is considered necessary for skin barrier function [8]. The lipid lamellae are organized by fully extended ceramides partly associated with cholesterol and FFAs [9]. Some cholesterol is also phase-separated within the lamellae [10]. In addition to the lamellar organization of the lipids, their lateral packing also affects skin permeability. The lipids in the SC layer are primarily packed in an orthorhombic or orderly and densely packed structure, with a smaller population forming a less densely packed hexagonal or liquid structure (Figure 1c,d) [8].

The corneocytes are surrounded by a highly cross-linked insoluble protein structure called the cornified envelope (CE). Specific lipid species, such as ω-hydroxy ceramides, fatty acids, and ω-hydroxy acids, bind covalently to the glutamate residues of the CE proteins, such as involucrin and loricrin, thus forming a lipid monolayer called the cornified lipid envelope (CLE) [11]. The CLE functions as a scaffold linking hydrophilic corneocytes and hydrophobic extracellular lipids (Figure 1c,e) [12,13]. Thus, the CLE is also considered essential for the permeability barrier function of the SC.

Abnormal composition and organization of the SC lipids can lead to a leaky barrier. Experimental evidence has demonstrated that epidermal lipid synthesis and processing are required to maintain skin barrier integrity [14]. In particular, ceramides synthesized in the epidermis play a critical role in the formation and function of the SC permeability barrier [15].

## 3. Diversity and Specificity of Epidermal Ceramides

**Figure 2:**
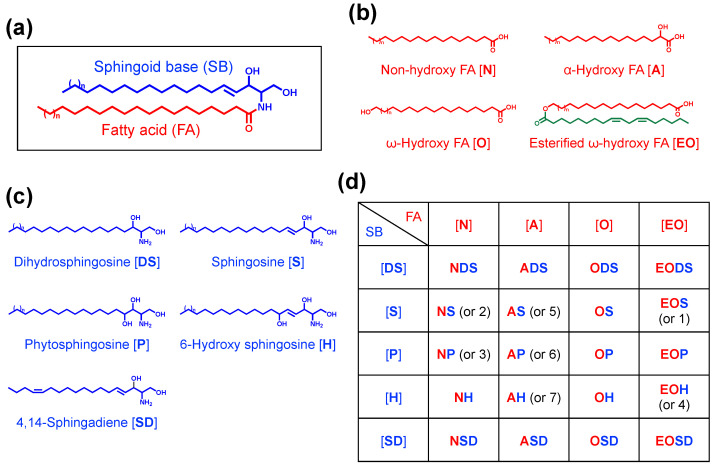
The structure and nomenclature of ceramides. (**a**) A ceramide is composed of a sphingoid base (SB) and a fatty acid (FA). (**b**,**c**) FAs (**b**) and SBs (**c**) in the major ceramide classes in human epidermis. (**d**) The different classes of ceramides are designated by a combination of the abbreviations of FAs and SBs. The numbers indicate their former numerical nomenclature.

A ceramide is composed of a sphingoid base (SB) and a fatty acid (FA), which are linked via an amide bond (Figure 2a). There are various classes of ceramides that result from the combination of different types of SBs and FAs. Approximately 20 years ago, at least seven ceramide classes were isolated from human SC via thin-layer chromatography (TLC) and were designated as ceramides 1–7 (Cer1–7). Studies using liquid chromatography–mass spectrometry (LC/MS) have identified several new classes of SC ceramides [16,17,18,19,20]. To date, each ceramide class is designated by a combination of the abbreviations of the FA and SB. The major ceramide classes in the epidermis are shown in Figure 2d. There are four types of FA—non-hydroxy [N], α-hydroxy [A], ω-hydroxy [O], and esterified ω-hydroxy [EO] FA—whereas there are five types of SB—dihydrosphingosine [DS], sphingosine [S], phytosphingosine [P], 6-hydroxy sphingosine [H], and 4,14-sphingadiene [SD] [16,20,21,22]. For example, a ceramide composed of non-hydroxy FA and sphingosine is represented as Cer[NS]. Other ceramide classes containing dihydroxy sphinganine [T] or β-hydroxy FA [B] are slightly present in human SC [22,23]. Rabionet et al. identified another new class of ceramides containing long acyl chains in both the *N*- and 1-*O*-positions, which are referred to as 1-*O*-acyl ceramides [24].

Although Cer[NS] exists in almost all tissues, ceramides containing esterified ω-hydroxy (EO) FA or 6-hydroxy sphingosine are unique to the epidermis [15,16,25]. There are several reports on the composition of unbound intercellular ceramides in human SC [20,22,23,26] (Table 1). In general, Cer[NP], Cer[NH], Cer[AH], and Cer[AP] are abundant in human SC, while EO ceramides account for 6.4–14.8% of total ceramides. Contrastingly, in mouse SC, more than half of the total ceramides are Cer[NS], which is followed by Cer[EOS] [22]. These data suggest that there are differences in ceramide metabolism in the epidermises of different species.

Each ceramide class includes various molecular species that vary in carbon chain length or degree of unsaturation. Several previous studies suggested that the FA chain length of human SC ceramides could vary between C14 and C36 [19,20]. Moreover, a recent comprehensive analysis of human SC ceramides revealed that in most ceramide classes other than EO ceramides, the primary type of FA composition was C26:0 or C24:0, whereas Cer[AS] predominantly contained C16:0 [22]. On the other hand, EO ceramides characteristically have saturated or monounsaturated FAs with 28 or more carbon chains. These FAs are occasionally referred to as ultra-long-chain FAs (ULCFAs), known to exist only in the epidermis [15,27]. Ceramides containing odd-chain FAs are also present in the SC [23]. There were no marked differences in the FA chain length between human and mouse SC [22]. In addition to FAs, SBs with different chain lengths also exist. Previously, most SC ceramides were thought to have an SB with 18 carbons [19]; however, Masukawa et al. [20] revealed that there were many ceramide species with long-chain (≥C18) SBs in human SC. In particular, SBs with long chain lengths (C24–C28) are frequently detected in Cer[NS] and Cer[NDS]. Due to the diversity of FAs and SBs, more than 1000 molecular species of ceramide exist in the epidermis [23].

Among epidermal ceramides, EO ceramides have unique structural features. EO ceramides contain ULCFAs (C28–C36) with a terminal ω-hydroxyl group that is esterified predominantly with linoleic acid (LA), an essential FA (Figure 2b). Based on their structure, EO ceramides are often referred to as ω-*O*-acylceramides or simply as acylceramides (acylCer). The presence of acylCer significantly contributes to the lamellar lipid organization in the SC [28]. Furthermore, acylCer is crucial for the formation of the CLE [13,15]. It is well known that a deficiency of LA, which is also known as essential FA deficiency, causes ichthyosis-like skin symptoms, with reduced skin barrier function. This deficiency is probably caused by the replacement of linoleate with oleate and other non-essential FAs [29,30]. Moreover, a recent study has demonstrated that LA serves as a natural substrate for the formation of the CLE [31]. Therefore, acylCer is an essential component of the epidermal permeability barrier.

## 4. Synthetic and Degradation Pathways of Epidermal Ceramides

**Figure 3:**
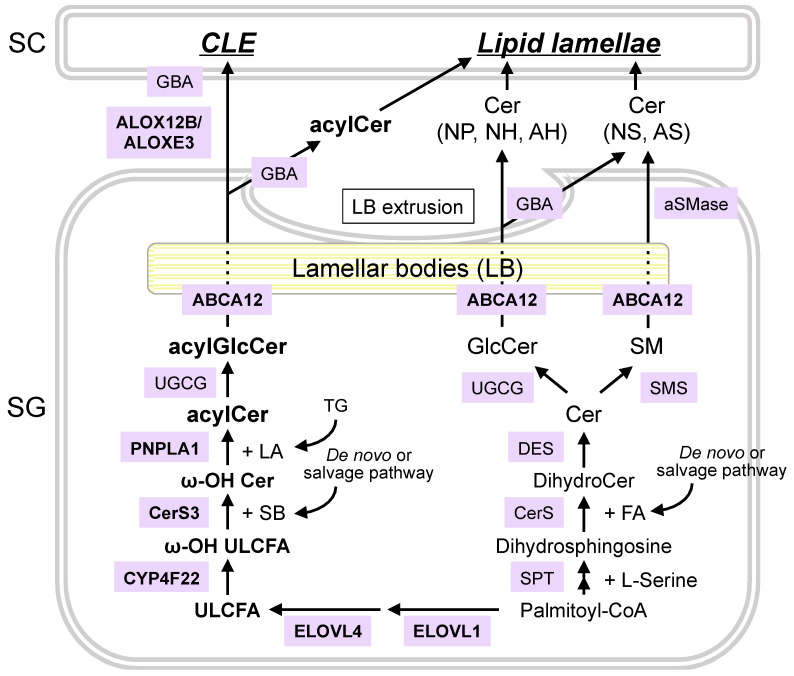
Synthetic pathways of epidermal ceramides (Cer). GlcCer, glucosylceramide; SM, sphingomyelin; ULCFA, ultra-long-chain fatty acid; acylCer, acylceramide; SB, sphingoid base; LA, linoleic acid; TG, triglyceride; SPT, serine palmitoyl-CoA transferase; CerS, ceramide synthase; DES, desaturase; UGCG, UDP-glucose ceramide glucosyltransferase; SMS, sphingomyelin synthase; ABCA12, ATP-binding cassette transporter 12; GBA, β-glucocerebrosidase; aSMase, acid sphingomyelinase; ELOVL, elongase of very long-chain fatty acid; CYP, cytochrome P450; PNPLA1, patatin-like phospholipase domain-containing 1; ALOX12B, 12*R*-lipoxygenase; ALOXE3, epidermis-type lipoxygenase-3.

Genetic and biochemical studies have revealed the entire synthetic pathway of ceramides in the epidermis (Figure 3) [15,27,32,33]. Ceramides are synthesized de novo in the epidermis, especially in the SG. The first step is the formation of 3-ketosphinganine by condensing L-serine and palmitoyl-coenzyme A (-CoA). This is catalyzed by serine palmitoyl-CoA transferase. The resulting 3-ketosphinganine is then reduced to sphinganine (or dihydrosphingosine) through the catalysis of 3-ketosphinganine reductase.

Dihydrosphingosine is then acylated by ceramide synthases (CerS) to form dihydroceramides. Non-hydroxy and α-hydroxy FAs are utilized to generate non-hydroxy and α-hydroxy ceramides, respectively. Furthermore, 2(α)-hydroxylation of FAs is catalyzed by the fatty acid 2-hydroxylase, encoded by the gene *FA2H* [34]. There are six mammalian CerS isoforms (CerS1–6), each of which has distinct substrate specificity toward acyl-CoA. For instance, CerS4 prefers stearoyl (C18)-CoA, arachidonoyl (C20)-CoA, and behenyl (C22)-CoA, while CerS3 has broad substrate specificity that exhibits activity toward acyl-CoA with ≥18 carbons [27].

Following *N*-acylation, dihydroceramides are converted to various types of ceramides by dihydroceramide desaturases (DESs). To date, two isoforms of DES have been characterized. The sphinganine moiety is desaturated to sphingosine by DES1 or hydroxylated to 4-hydroxy sphinganine (i.e., phytosphingosine) by DES2 [35]. However, the enzyme responsible for converting the sphinganine moiety to 6-hydroxy sphingosine has not yet been identified.

In addition to de novo synthesis, salvage pathways are also critical for epidermal ceramide synthesis. Newly synthesized ceramides are converted to glucosylceramides (GlcCer) or sphingomyelin (SM) in the Golgi apparatus, which are then incorporated into lamellar bodies (LBs), which proliferate in the late stages of epidermal differentiation. ATP-binding cassette transporter 12 (ABCA12) plays a crucial role in the transport of lipids into LBs [36]. Subsequently, LBs fuse with the apical plasma membrane of the SG, which is then followed by the secretion of their contents at the SG and SC interface. The released GlcCer and SM are hydrolyzed back to ceramides by β-glucocerebrosidase (GBA) and acid sphingomyelinase (aSMase) to form the SC lipid lamellae. Studies have demonstrated that GlcCer is the primary precursor of all ceramides except acylCer, whereas SM generates only Cer[NS] and Cer[AS] [37,38].

**Figure 4:**
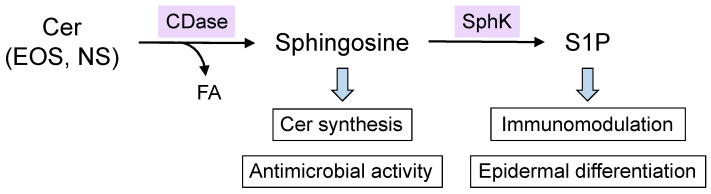
Degradation pathway of ceramides and function of ceramide metabolites. Sphingosine-containing ceramides, such as Cer[EOS] and Cer[NS], are degraded by ceramidase (CDase), which yields a fatty acid (FA) and sphingosine. The resulting sphingosine is metabolized to sphingosine-1-phospate (S1P) by sphingosine kinase (SphK). These ceramide metabolites have various physiological functions, as indicated in the boxes.

Eventually, the ceramides are hydrolyzed into SBs and FAs by ceramidase (CDase) (Figure 4). There are five CDase isoforms (acid CDase, neutral CDase, and alkaline CDase 1–3) with different pH optima and substrate specificities in mammals, all of which are expressed in the epidermis [39]. The resulting SBs are then re-utilized as substrates for ceramide synthesis. For example, Cer[NS] can produce other ceramide species, such as Cer[EOS]. Since mammals cannot directly convert dihydrosphingosine to sphingosine, sphingosine is generated only by degrading ceramides containing sphingosine. Therefore, salvage or recycling pathways can contribute to the diversity of epidermal ceramides.

The unique structural features of acylCer indicate that their biosynthesis involves multiple enzymatic reactions, which include (1) FA elongation, (2) ω-hydroxylation, (3) *N*-acylation, and (4) ω-*O*-esterification.

In FA elongation, very long chain FAs are synthesized from palmitoyl-CoA on the endoplasmic reticulum membrane through the catalysis of FA elongases (ELOVL). In mammals, seven ELOVL isoforms (ELOVL1–7) exist. Each isoform has a specific substrate specificity and physiological roles. In the epidermis, ELOVL1 plays a pivotal role in the production of hexacosanoyl (C26)-CoA, which is further elongated to acyl-CoA with ≥28 carbons by ELOVL4. Thus, both enzymes are required for the generation of acylCer with ULCFAs (C28–C36).

In ω-hydroxylation, ULCFAs are ω-hydroxylated to form ω-hydroxy ULCFAs after the removal of CoA. The enzyme that catalyzes this reaction has recently been identified as cytochrome P450, family 4, subfamily F, polypeptide 22 (CYP4F22) [40].

In *N*-acylation, after the conversion to ω-hydroxy ultra-long-chain acyl-CoA, CerS3 exclusively catalyzes the acylation of SBs, which are derived from the de novo or salvage pathways, to generate ω-hydroxy ceramides. CerS3 has a wide range of substrate specificity, thereby enabling the use of ω-hydroxy ultra-long-chain acyl-CoA as a substrate. Then, the SB moiety of ω-hydroxy ceramides is converted to other types of SBs by the corresponding DES, as described above.

Finally, in ω-*O*-esterification, ω-hydroxy ceramides are esterified with LA to generate acylCer. The responsible enzyme has recently been identified as patatin-like phospholipase domain-containing protein 1 (PNPLA1) [41]. PNPLA1 functions as a transacylase that catalyzes ester formation using triglycerides as the donor of LA. A recent study has shown that α/β-hydrolase domain-containing 5, known to be involved in ω-*O*-esterification, interacts directly with PNPLA1 and stimulates PNPLA1-mediated acylCer generation [42].

The generated acylCer is converted to acylGlcCer in the Golgi bodies. This is followed by the incorporation of acylGlcCer into LBs by ABCA12. AcylGlcCer released from the LBs is partly reverted to acylCer by GBA, which then forms extracellular lipid lamellae. In contrast, acylGlcCer is the primary source of the CLE. The linoleate moiety of acylGlcCer is hydrolyzed by the sequential actions of two lipoxygenases, 12*R*-lipoxygenase ALOX12B and epidermal lipoxygenase-3 ALOXE3, which results in reverting to ω-hydroxy ceramides [31,43]. Subsequently, the exposed ω-hydroxyl group is cross-linked to cornified envelope proteins, which then forms the CLE.

Notably, mutations in genes involved in acylCer generation (ELOVL1, ELOVL4, CYP4F22, CERS3, and PNPLA1) and CLE formation (ALOX12B and ALOXE3) have been reported to cause similar phenotypes characterized by severe skin barrier defects in humans and mice [40,41,44,45,46,47,48,49,50,51,52]. These findings emphasize the unique importance of acylCer and the CLE in epidermal barrier formation and function.

Ceramide metabolites, such as sphingosine and sphingosine-1-phosphate (S1P), have diverse physiological functions. In terms of skin physiology, sphingosine has broad-spectrum antimicrobial properties [53]. In addition, S1P modulates immune responses and promotes epidermal differentiation (Figure 4) [54].

## 5. Altered Amount and Composition of Ceramides and Other Lipids in the Skin of Patients with AD

The epidermal barrier is essential for maintaining skin homeostasis, and the disruption of its function is often associated with various skin diseases. AD is the most common inflammatory skin disease, with a lifetime prevalence of up to 20% [55]. Moreover, its incidence has increased in industrialized countries over the years [56]. Hence, it is important to know the pathogenesis of AD to develop effective therapeutic modalities to reduce its incidence. In AD, the skin barrier is dysfunctional. Since SC lipids have been associated with skin barrier function, SC lipid changes in patients with AD have been extensively investigated. This section summarizes the findings on the alteration of lipids, especially ceramides, in AD skin.

Early studies used TLC to determine the amount and composition of extractable or unbound intercellular SC lipids in patients with AD. Imokawa et al. and others showed that total ceramide levels were significantly reduced in patients with AD compared with those in healthy controls [57,58]. Cholesterol levels remained the same or increased in patients with AD [58,59,60]. Although ceramides, cholesterol, and FFAs are present at an equimolar ratio in a normal SC, the ceramide/cholesterol ratio was significantly reduced in AD cases [59]. Several studies have consistently shown that Cer1 (i.e., Cer[EOS]) levels were markedly decreased in AD cases [57,58,59,60,61]. Yamamoto et al. further analyzed the FA composition of Cer1 using gas chromatography and found that the levels of Cer1 esterified with C18:1 (i.e., oleate) were increased in AD patients [60]. This suggests that acylCer is also functionally abnormal in AD. Furthermore, Di Nardo et al. found that Cer3 (i.e., Cer[NP]) was also reduced, which was negatively correlated with transepidermal water loss (TEWL), a parameter of SC barrier function [59].

Later studies using LC/MS further elucidated the alterations in intercellular SC ceramides in AD. Ishikawa et al. [62] reported that Cer[EOS], Cer[EOH], Cer[EOP], Cer[NP], and Cer[NH] were decreased in patients with AD, whereas Cer[AS] was increased in the SC of patients with AD. These ceramide changes were correlated with increased TEWL. They also found that the total carbon number of ceramides was altered in patients with AD. Although ceramides with a total carbon number lower than 40 are rarely found in a normal SC, smaller species, such as those with a total carbon of 34 (C34 Cer), are characteristically increased in AD patients, whereas larger species or those with more than 50 total carbons are decreased. C34 Cer[NS] levels were significantly correlated with increased TEWL. Bouwstra et al. also clarified that such changes in SC ceramides were more markedly observed in the skin lesions than in the normal skin of AD patients [63,64,65]. Other studies have confirmed that similar changes in SC ceramides occur in AD [66,67,68].

Macheleidt et al. [69] first reported the alteration in protein-bound lipids in AD. They used epidermal samples to analyze the lipids in the SC using TLC and found that the amount of protein-bound ceramides was more markedly decreased in lesional skin compared with that in non-lesional skin. However, a different finding was recently reported by Boien et al., who used tape-stripped SC samples and analyzed the bound ceramides using LC/MS. In this study, total bound ceramides did not differ between healthy controls and AD patients, while bound ceramides with unsaturated FAs or sphingosine were conversely increased in AD patients [70].

The composition of FFAs is also altered during AD. Several studies showed that although very long-chain FFAs (≥24 carbons) were reduced in AD, shorter FFAs (especially palmitic acid (C16:0) and stearic acid (C18:0)) and unsaturated FFAs were increased in patients with AD [64,69]. These changes in the FFA profile were more frequently observed in lesional skin compared with non-lesional skin.

Altered lipid composition may contribute to abnormal SC lipid organization. Analysis of SC lamellar organization using small-angle X-ray diffraction revealed that lipid lamellae with LPP were absent in AD patients with reduced levels of acylCer [63,71]. Thus, the abnormal lamellar organization in AD may be attributed to the lack of acylCer.

Lateral lipid organization is affected by the FFA composition. Studies using electron diffraction or Fourier transform infrared spectroscopy showed that a less dense, hexagonal organization, instead of a highly dense orthorhombic organization, was frequent in the SC of AD patients. Notably, the reduced orthorhombic structure was well correlated with the mean chain length of the FFA [63,64].

**Figure 5:**
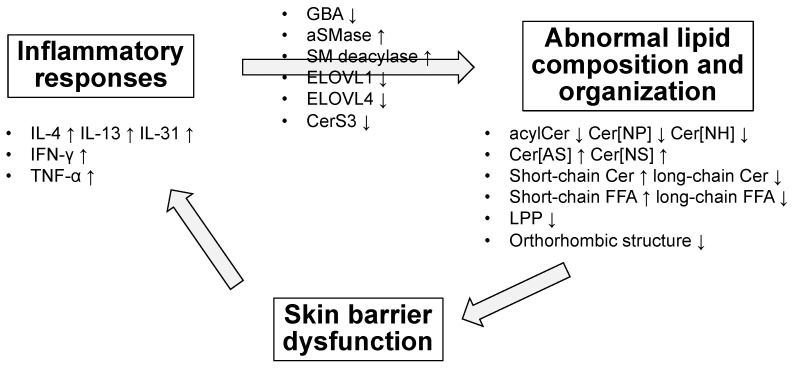
Lipid abnormalities in atopic dermatitis (AD) and their possible underlying mechanisms. In patients with AD, the composition and organization of lipids, especially ceramides, are altered; thus, the expression and activities of the enzymes associated with ceramide synthesis or degradation are involved. The mechanism has not been fully elucidated, but the involvement of Th2 and Th1 cytokines and that of TNF-α are implicated.

As noted above, AD skin is associated with lipid abnormalities (Figure 5). Collectively, the extractable SC lipids, such as acylCer, Cer[NP], and Cer[NH], are reduced, whereas Cer[AS] and Cer[NS] are increased [57,58,59,60,61,62,63,64,65,66,67,68]. In addition, short-chain rather than long-chain ceramides are increased [62,63,64,65]. Such a shift toward ceramides with a shorter chain length is also observed in FFAs [64,69], raising the hypothesis that SC ceramides and FFAs share a common synthetic pathway. Along with these compositional changes, the densely packed, LPP lamellar structure is reduced [63,64,71]. There are conflicting reports on the changes in bound ceramides in AD [69,70].

## 6. Possible Causes and Mechanisms of Lipid Abnormalities in AD

Since the amount and composition of ceramides and FFAs are typically altered in AD, changes in the expression and activities of enzymes involved in their synthesis or degradation are involved. This section summarizes the possible factors that determine lipid abnormalities in AD (Figure 5).

As GBA is the primary enzyme for SC ceramide production, defects in this enzyme may occur in AD. Cui et al. reported that the amount of precursor protein of the activator protein of GBA (prosaposin) was decreased in patients with AD [72]. However, Jin et al. reported that GBA activity in the SC was unchanged in patients with AD [73]. On the other hand, Boer et al. recently examined the localization of GBA activity by using an activity-based probe in the epidermis and found that GBA activity was less localized in patients with lesional AD skin than in the normal controls. Notably, the altered location of GBA activity was correlated with a decrease in the overall SC ceramide levels [74].

Kusuda et al. previously examined the localization of aSMase by immunostaining. Immunoreactivity for aSMase was observed from the upper spinous cell layer to the upper SC and slightly increased in the lesional skin of patients with AD [75]. Jensen et al. reported that the enzymatic activities of aSMase were decreased in AD skin [76]. However, this report did not account for the global reduction in SC ceramides observed in patients with AD because it could produce only Cer[NS] and Cer[AS]. On the other hand, Boer et al. recently examined the localization of aSMase using in situ zymography and found that aSMase was present or increased in the whole SC of lesional AD skin, which was correlated with an increase in the total amounts of Cer[AS] and Cer[NS] [74].

There are various findings and speculations regarding CDase and ceramides in AD skin. First, the enzymatic activity of alkaline CDase remained unchanged in AD cases [73]. Second, acid CDase was downregulated in AD, thus leading to a decrease in the level of antimicrobial sphingosine [77,78]. Third, several bacterial species in AD skin secreted CDase, which resulted in ceramide degradation [79].

Imokawa et al. [80] stated that a novel enzyme was involved in ceramide deficiency in patients with AD. Activity of a previously unknown enzyme was observed in AD skin, which was referred to as SM deacylase [81]. This enzyme cleaved the *N*-acyl linkage of SM and GlcCer, which then resulted in either glucosyl sphingosine or sphingosyl phosphorylcholine. SM deacylase was not identified in the epidermis and was thought to be of bacterial origin. However, a recent study revealed that the amino acid sequence of SM deacylase was identical to the β-subunit of acid CDase [82].

In AD, lipid abnormalities are more evident in lesional skin than in non-lesional skin. Th2 immune responses characterize acute skin lesions, while Th1 responses are involved in chronic lesions. Therefore, these inflammatory cytokines may contribute to the lipid abnormalities in AD.

In an in vitro study, Hatano et al. showed that the Th2 cytokine interleukin-4 (IL-4) suppressed GBA and aSMase expression and ceramide synthesis induced by the pro-inflammatory cytokine tumor necrosis factor-α (TNF-α) and the Th1 cytokine interferon-γ (IFN-γ) in the human epidermis [83]. The same group also showed that IL-4 delayed barrier recovery after acute disruption in mice [84]. Park et al. found that the expression of *Elovl1* and *Elovl4* decreased concomitantly with decreased levels of ceramides with very long chain FAs in oxazolone-treated AD model mice [85]. In human skin equivalents, Danso et al. examined the effects of cytokines on SC lipid properties and organization. They found that TNF-α and Th2 cytokines (IL-4, IL-13, and IL-31) decreased the levels of very long chain FFAs and acylCer, which consequently affected lipid organization. The protein levels of ELOVL1 and CerS3 were markedly reduced by treatment with these cytokines [86]. Interestingly, the same group showed that the expressions of GBA, aSMase, ELOVL1, and CerS3 were also altered in the lesional skin of AD patients [65]. On the other hand, a recent study by Berdyshev et al. showed that the expression levels of *ELOVL3* and *ELOVL6* were significantly decreased in the skin of human AD patients and IL-13 transgenic AD model mice, although *ELOVL1* and *ELOVL4* were increased [87].

The canonical Th1 cytokine IFN-γ also affected the lipid barrier components. Sawada et al. used human epidermal equivalents to show that SC ceramide levels were slightly increased after IFN-γ treatment [88]. In addition, Tawada et al. examined the effects of IFN-γ on SC lipid profiles using cultured human keratinocytes and epidermal sheets. They found that IFN-γ decreased the level of ceramides with long-chain FAs. This possibly occurred through the downregulation of ELOVL1 and CerS3 [89]. Consistently, long-chain ceramides and their related enzymes (Elovl1, Elovl4, and CerS3) were reduced in the skin of house dust mite allergen-induced AD model mice [90].

Evidence has recently accumulated that skin barrier defects play a primary role in AD. For example, common loss-of-function mutations in filaggrin (FLG), a keratin filament-aggregating protein, are the major important predisposing factors for AD [91]. Several studies have investigated the effects of FLG mutations on SC barrier lipids. Although a study showed that the levels of Cer[EOH] and triglycerides were more reduced in patients with AD with FLG mutations than in those with wild-type AD [92], other studies did not find any association between FLG mutations and SC lipid alterations [63,93]. However, FLG is downregulated by Th2 cytokines, regardless of FLG mutations, leading to barrier lipid abnormalities through multiple pathways [94].

## 7. Potential Therapeutic Approaches to Correct Lipid Abnormalities in Patients with AD

Currently, topical corticosteroids and topical calcineurin inhibitors are widely used for the treatment of AD. Both drugs have been reported to exert adverse effects on the skin barrier homeostasis of normal skin [95,96]. On the contrary, these drugs can improve the epidermal barrier function in long-term treatment of patients with AD [97]. A recent study also showed that 12-week proactive treatment with tacrolimus increased ceramide levels and normalized intercellular lipid lamellae [98]. This controversy may be due to their anti-inflammatory effects, which compensate for the adverse effects on skin barrier function. Dupilumab and delgocitinib, which were recently approved for the treatment of AD, specifically inhibit IL-4/IL-13-mediated signaling. Thus, these drugs may prevent inflammation-induced barrier dysfunction more effectively than other conventional topical corticosteroids [99,100].

**Figure 6:**
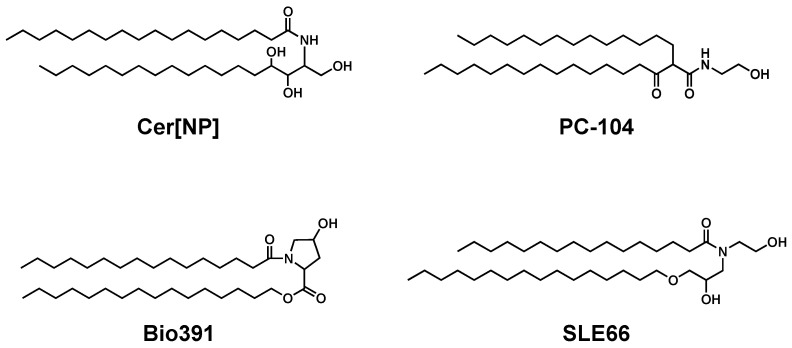
Chemical structure of natural and skin-identical ceramide (Cer[NP]) and synthetic pseudo-ceramides. These structures are cited from the literature [111,113].

In addition to anti-inflammatory therapies, strategies to directly restore skin barrier function have been found to be promising for the treatment of AD. Previous animal studies showed that although topical application of ceramide alone delayed barrier recovery, lipid mixtures of ceramides, cholesterol, and FFAs accelerated barrier recovery. Subsequently, a ceramide-dominant lipid mixture with a 3:1:1 molar ratio was shown to be ideal for barrier restoration [101]. Several clinical studies have shown that ceramide-dominant emollients represent a safe and useful adjunct to AD treatment [102,103]. Many other studies have shown the beneficial effects of ceramide-containing emollients on skin condition in xerosis and AD [104,105,106,107,108,109,110] (partly summarized in Table 2). Several of these studies also demonstrated that the lipid lamellar structure and ceramide composition in the SC were normalized [102,109]. As natural and skin-identical ceramides are expensive, various synthetic pseudo-ceramides have been developed and tested [111,112,113] (Figure 6). Various formulations have also been developed to enhance ceramide bioavailability, including those with liposomes and multi-vesicular emulsions [104,106,114,115]. A recent clinical study showed that a cream or lotion containing ceramides in a multi-vesicular emulsion could sustain skin moisturization longer than commonly used traditional emollients [109]. Interestingly, a large randomized controlled trial is currently underway to determine whether routine prophylactic use of ceramide-dominant emollients can prevent the onset of AD and the subsequent allergic march associated with those with AD [116].

Alternatively, certain polyunsaturated fatty acids (PUFAs) may have beneficial effects on epidermal barrier function. As described above, it has been shown that a deficiency of n-6 PUFA LA causes skin barrier defects, probably by decreasing acylCer-mediated CLE formation. We have reported that a dietary deficiency of both unsaturated fatty acids and starch causes AD-like symptoms with skin barrier defects in mice [117,118]. Using the diet-induced AD mouse model, we have shown that LA metabolites, such as γ-linolenic acid and arachidonic acid, restore skin barrier dysfunction and ameliorate AD symptoms [119]. Furthermore, we recently found that an n-3 PUFA, eicosapentaenoic acid, can also correct barrier abnormalities, probably by restoring covalently bound ceramides in the SC [120]. However, the detailed mechanism by which these PUFAs correct ceramide abnormalities and restore skin barrier function remains unknown.

## 8. Conclusions

Many studies have demonstrated that AD skin has an abnormal composition of barrier lipids, especially ceramides. Given the central role of ceramides in skin barrier formation, ceramide abnormalities are involved in the pathogenesis of AD. The mechanism underlying ceramide abnormalities in AD has yet not been fully elucidated, but the involvement of Th2 and Th1 cytokines is implicated. Preventing and restoring skin barrier dysfunction are the cornerstones of the management of AD. Exogenously applied ceramide-dominant emollients have beneficial effects on skin barrier function accompanied with normalization of abnormal ceramide composition. Furthermore, various ceramide-containing formulations have been developed. If topical ceramide application can effectively prevent the onset and exacerbation of AD, it may be possible to minimize the use of corticosteroids. Drugs that intrinsically correct ceramide abnormalities might also be desirable as a barrier repair agent of AD. Further elucidation of the mechanism of lipid abnormalities in AD can provide important implications for the development of new therapeutic approaches for the treatment of AD.

## Figures and Tables

**Table 1:** The composition of unbound intercellular ceramides in human SC.

	NDS	NS	NP	NH	NSD	NT	ADS	AS	AP	AH	ASD
Masukawa et al. [20]	5.9	6.3	21.3	22.6	n.d.	n.d.	0.8	3.6	16.1	15.7	n.d.
t’Kindt et al. [23]	9.8	7.4	22.1	14.5	n.d.	1.7	1.6	9.6	8.8	10.8	n.d.
van Smeden et al. [26]	8.6	6.7	25.8	12.4	n.d.	n.d.	1.9	3.8	13.4	12.4	n.d.
Kawana et al. [22]	6.2	5.2	24.2	23.7	0.1	n.d.	0.9	4.3	9.2	18.0	0.2
	**ODS**	**OS**	**OP**	**OH**	**OSD**	**BS**	**EODS**	**EOS**	**EOP**	**EOH**	**EOSD**
Masukawa et al. [20]	n.d.	n.d.	n.d.	n.d.	n.d.	n.d.	n.d.	4.3	0.9	2.5	n.d.
t’Kindt et al. [23]	n.d.	0.7	0.2	0.4	n.d.	n.d.	0.4	6.5	1.1	4.3	n.d.
van Smeden et al. [26]	n.d.	n.d.	n.d.	n.d.	n.d.	n.d.	1.3	5.4	2.7	5.4	n.d.
Kawana et al. [22]	0.1	0.6	0.3	0.6	0.0	0.2	0.1	2.1	1.0	3.1	0.0

The data were obtained from the literature [20,22,23,26]. The values represent the mean percentage of total ceramides. The color indicates the scale from trace (white) to abundant (red). n.d. means not determined.

**Table 2:** Clinical studies examining the effects of ceramide-containing emollients on xerosis and AD.

Ceramide Species (Compound Name)	Formulation (Brand Name)	Outcome	Reference
Pseudo-ceramide (PC-104)	Cream (Triceram^®^)	Disease severity ↓ TEWL ↓ SC hydration ↑ Extracellular lipid lamellae ↑	Chamlin et al. [98]
Pseudo-ceramide (PC-104)	Cream (Epiceram^®^)	Disease severity ↓ pruritus ↓ sleep ↑	Sugarman et al. [99]
Pseudo-ceramide (SLE66)	Cream (Curel^®^)	Disease severity ↓ TEWL → SC hydration ↑	Seghers et al. [103]
Pseudo-ceramide (PC-104)	Cream (NeoCera™)	Disease severity ↓ TEWL → SC hydration ↑	Draelos et al. [107]
Pseudo-ceramide (SLE66)	Oil-in-water lotion	Disease severity ↓ TEWL ↓ SC hydration ↑ Cer[NH], Cer[NP] ↑ Cer[NS], Cer[AS] ↓	Ishida et al. [108]
Cer[NP], Cer[AP], Cer[EOS]	Multivesicular emulsion (CeraVe^®^)	SC hydration ↑ Skin dryness ↓	Danby et al. [109]

## Abbreviations

ABCA12, ATP-binding cassette transporter 12; acylCer, acylceramide; AD, atopic dermatitis; ALOX12B, 12*R*-lipoxygenase; ALOXE3, epidermal lipoxygenase-3; aSMase, acid sphingomyelinase; CDase, ceramidase; CE, cornified envelope; Cer, ceramide; CerS, ceramide synthase; CLE, cornified lipid envelope; CoA, coenzyme A; CYP, cytochrome P450; DES, desaturase; ELOVL, fatty acid elongase; EO, esterified ω-hydroxy; FA, fatty acid; FFA, free fatty acid; FLG, filaggrin; GBA, β-glucocerebrosidase; GlcCer, glucosylceramide; IFN, interferon; IL, interleukin; LA, linolenic acid; LB, lamellar body; LC/MS, liquid chromatography–mass spectrometry; LPP, long periodicity phase; PNPLA1, patatin-like phospholipase domain-containing protein 1; PUFA, polyunsaturated fatty acid; S1P, sphingosine-1-phosphate; SB, sphingoid base; SC, stratum corneum; SG, stratum granulosum; SM, sphingomyelin; SPP, short periodicity phase; TEWL, transepidermal water loss; TLC, thin-layer chromatography; TNF, tumor necrosis factor; ULCFA, ultra-long-chain fatty acid.
